# Harmonizing lecture slides presentations with background music: thematic analysis of students experiences

**DOI:** 10.3389/fpsyg.2025.1637073

**Published:** 2025-11-13

**Authors:** Gyasi Alfred Bannor, Yarhands Dissou Arthur

**Affiliations:** Department of Mathematics Education, Akenten Appiah-Menka University of Skills Training and Entrepreneurial Development, Kumasi, Ghana

**Keywords:** background music (BM), concentration, lecture slide presentations, learning retention, PowerPoint presentation, University, Ghana

## Abstract

Research shows that background music (BM) help people stay engaged and focused while learning, reading, or performing other cognitive tasks, though it can sometimes be distracting. However, its role in learning during lecture slide presentations is not well understood. This study explores the impact of BM on learning during PowerPoint lecture presentations among a convenient sample of 23 Master of Philosophy (MPhil) students in Ghana. Using a descriptive phenomenological qualitative design, a semi-structured interview was employed to elicit students’ responses of their experiences. Thematic analysis was conducted of the interview data and three main themes were identified: (i) staying engaged, where BM made the learning environment livelier, (ii) effect on concentration, with mixed responses on whether BM helped or distracted attention, and (iii) effects on learning, where BM supported some students’ understanding, but hindered that of others. The findings suggest that BM’s impact on learning during slide presentation context is influenced by individual factors. That is, the effects of BM may differ based on individual or contextual factors. Thus, we recommend the need for lecturers to consider individual students regarding integrating BM into PowerPoint presentations.

## Introduction

Music has been an integral part of many people’s lives. It is often described as “the wine which inspires one to new generative processes” (Doyle and Furnham, 2012). In recent years, its role in cognition has attracted significant attention, but with mixed findings (de la Mora Velasco et al., 2023). Despite the growing interest in this area, there remains a gap in research specifically exploring the effects of background music (BM) in university-level lectures slide deck presentations. This lack of research highlights the need for further investigation into the potential cognitive benefits or drawbacks of BM in this common instructional context.

BM research has revealed generally inconclusive effects on learning and cognition in language and mathematics. Kämpfe et al. (2010) concluded that BM typically exerts small detrimental or negligible effects on memory performance of adult listeners. de la Mora Velasco and Hirumi (2020) found inconclusive effects but indicated that benefits for fact memorization were rare, with disruptions more common. Lehmann and Seufert (2017) and Echaide et al. (2019) revealed that between BM and recall capacity there is no effect. Que et al. (2023) found no significant effect of BM on passage comprehension accuracy or metacognition. Taheri et al. (2022) showed insignificant effects of BM on attention among students aged 18–30 years. BM has little to no benefit on recall, as it does not significantly accelerate recall (Karimnia and Lari, 2012), or improve word retrieval (Ferreri et al., 2015), and it may even disrupt writing fluency (Ransdell and Gilroy, 2001). Others find no significant impact on exam results (Abdullah et al., 2022; Chen and Wen, 2015).

Further complicating the picture, patterns of BM’s potential benefits appear to be highly context-dependent and vary based on music tempo, volume, preferences, personality, and cognitive task and even with this, effects are found to be at best, neutral or negligible; at worst, mildly disruptive. Cheah et al. (2022) reported that BM particularly when it included lyrics, tended to interfere with tasks involving memory and language processing. Salamé and Baddeley (1989) showed that vocal music disrupted phonological short-term memory, while purely instrumental music was less disruptive but still failed to enhance performance. Pring and Walker (1994) similarly observed that instrumental music might be less harmful than lyrical music on short term memory, yet provided no evidence for a genuine improvement in memorization. Fast and loud BM was found to disrupt reading comprehension (Thompson et al., 2011), and stimulating BM hinder arithmetic tasks (Hallam et al., 2002). More recently, Lin et al. (2023) found that slowing tempo music does not systematically improve cognitive performance and, in some cases, may actually worsen it by reducing processing speed.

On the other hand, there are studies that had reported positive effects of BM in cognitive tasks, although context matters. BM has been found to enhance writing fluency, improve essay quality (Ransdell and Gilroy, 2001), and boost test scores and accuracy rates in specific contexts (Guo et al., 2021). Some studies report improved performance in mathematics tasks, such as enhanced trigonometry test scores under Mozart’s music (Taylor and Rowe, 2012). Hallam et al. (2002) found that calming and relaxing BM enhances arithmetic performance and memory task among children aged 10–12. Kiss and Linnell (2021) identified preferred BM enhance task-focused attention. Cournoyer (2019) found out that students could memorize three word lists faster in the presence of stimulating or relaxing BM which shows that episodic memory could benefit from the presence of stimulating BM. Anderson et al. (2000) indicated an improvement in word spelling and word retention. The study showed that BM enabled the students to concentrate, relax and revisualize in the spelling of the words. Hofbauer et al. (2024) identified that fast tempo music correlated with improved cognitive performance with differences observed individual wise. Moreover, the study by Du et al. (2020) demonstrated that in their experiment, students under BM condition showed higher N400 effect since they experienced higher brain effort to process meaning from words than those put under the no-BM condition.

These mixed findings shows that there are inconsistencies which suggest that BM’s effects have not been universal across learning contexts and that BM’s role is complex in learning and cognitive tasks and hence the need for more understanding. Meanwhile, BM’s specific role in multimedia learning environments, such as slide deck presentations, remains underexplored.

Slide deck presentations are common instructional tools at universities but it often fails to fully engage learners when used as a standalone medium (Clark, 2008; Ferreira et al., 2018; Strauss et al., 2011). Research suggests that synchronizing slides with BM may help manage cognitive load and enhance focus (e.g., Griffin et al., 2009), yet empirical investigations into BM’s effects while learning with slide deck presentations remain scarce. The purpose of this study was to investigate the experiences of students regarding how background music (BM) influences their learning experiences during lecture slide presentations. The aim was to understand the effect of BM on students’ overall learning: concentration, attention, and cognition.

The use of BM in slide presentations may align with principles of multimedia learning where auditory and visual stimuli work together to create an immersive learning experience (Gerdes et al., 2014; Shams and Seitz, 2008). When discussing slides synchronized with music, we considered expanding this idea based on two key works: (1) Moreno and Mayer (2000) and (2) de la Mora Velasco and Moreno (2025). Moreno and Mayer (2000) describe BM as a “seductive detail” in multimedia learning. Moreno and Mayer (2000) believe that BM often acts as something that makes a presentation more entertaining or attractive to audience but can pull attention away from the main instruction content or ideas. According to this description, every aspect of multimedia instruction should serve a clear learning purpose, and music that does not directly support such purposes should not risk getting in the way. On the other hand, de la Mora Velasco and Moreno (2025) offer a more optimistic view. de la Mora Velasco and Moreno (2025) propose that music can be used in ways that avoid split attention or cognitive overload, such as when it is strategically integrated. In this view, when music is carefully chosen and aligned so that it matches the tone, pacing, or motion of the instruction delivery, it can actually enhance engagement and make learning more interesting. In this sense, BM does not seem certainly good or bad; it depends on how it is integrated. When thoughtfully integrated, it can create rhythm, focus attention, and bring emotional coherence to learning. But when used carelessly, it can just as easily distract since auditory information can sometimes overshadow visual processing (Echaide et al., 2019). This raises critical questions about whether BM might complement or compete with visual content in slide presentations. These questions are particularly relevant given the inconsistent effects of BM observed across various learning contexts (e.g., de la Mora Velasco et al., 2023).

Rehn (2016) wrote in the *Art of Keynoting* that attention spans during slide presentations using speech-based narration typically last only 5–10 min, after which the engagement of the audience declines. For this matter, we argue that BM with its ability to evoke emotional and cognitive responses (Koelsch, 2014; Piccardi et al., 2024), may offer a potential solution to this challenge by fostering sustained attention and enriching the overall learning experience. However, given the mixed findings of BM’s effects in cognitive tasks and learning environments, it might be very difficult to conclude either it has the potential to either support or detract from visual content in slide presentations. Hence, there is a pressing need for in-depth qualitative study. Such research would clarify whether BM enhances or disrupts the cognitive processes involved in slide presentations and contribute to a broader understanding of its role in multimedia learning contexts of this sort. Accordingly, this study employs descriptive phenomenological qualitative design to investigate the perceptions of 23 Master of Philosophy (MPhil) students who attended lectures featuring PowerPoint presentations accompanied by calming Gospel music song by Don Moen. The sample size of 23 was deemed sufficient since data saturation may have occurred on the 24th interviewee. According to Guest et al. (2006), data saturation is said to have occurred when no new insights emerge from interviews. Hence, we believe that with this sample size we adequately captured the diversity and depth of participants’ experiences. The study aims to contribute new insights into the ongoing debate on BM’s role in multimedia learning and provide practical implications for lecturers seeking to optimize instructional strategies utilizing BM.

## Materials and methods

### Participants

Recruitment of participants were done by sending invitation links developed with Google forms through social media posts, specifically through WhatsApp platforms. By clicking the link, participants could provide contact details or any other information confirming their availability for the study. Participants who met the inclusion criteria of having “no hearing impairments” were then invited to participate in the study at their own consent. A total of 23 MPhil students took part in the study and the interview process. This sample was chosen through convenience sampling procedure. The sample size was deemed adequate by the principles of data saturation which may have occurred after the 24th interviewee, as suggested by Guest et al. (2006). Of these 23 participants, 20 were male and 3 were female. Participants ages ranged from 28 to 46 years, with an average age of 37. The participants comprised students from different religious backgrounds, with 14 identifying as Christians and 9 as Muslims. In terms of marital status, 12 participants were married, 2 were divorced, and 9 were single.

### Materials and measures

In this study, PowerPoint slides, a song and an interview guide were used. The PowerPoint slides were designed to deliver content on multivariate statistics, focusing on key concepts such as factor analysis, multiple regression, and principal component analysis. The slides incorporated text, diagrams, and graphs to aid in the visualization of complex statistical concepts, and were enhanced with BM. This allowed to investigate its potential impact on the learning experience. The song “Our Father” by Don Moen^1^ was used as the BM during the PowerPoint presentation. Following the framework of de la Mora Velasco and Moreno (2025) for reporting the characteristics of music, the song can be described as a contemporary Christian worship song that feature vocals and lyrics derived from The Lord’s Prayer (“Our Father who art in heaven…”). It is set in a major key with a slow to moderate tempo (approximately 70–80 BPM) and a regular 4/4 meter, which produced a calm and reverent atmosphere. The instrumentation includes piano, acoustic guitar, soft percussion, and vocal harmonies, with the volume maintained at a moderate level (around 60–65 dB) to ensure clarity without overpowering other auditory inputs. The slides were carefully structured to ensure clarity and to facilitate the flow of information, with each slide designed to introduce concepts progressively, followed by examples and exercises for reinforcement. The semi-structured interview guide, was used to explore participants’ experiences of the PowerPoint lectures with BM. The interview guide consisted of open-ended questions designed to encourage reflection and provide rich qualitative data. Questions focused on the participants’ engagement with the material, the perceived impact of the background music on their attention and retention, and how they felt the music affected their overall learning experience. The interview guide was designed to be flexible, allowing for follow-up questions and exploration of unexpected themes that arose during the interviews.

### Procedures

(1) Ethical approval was obtained for the study from the ethics committee of the School of Graduate Studies of the Akenten Appiah-Menka University of Skills Training and Entrepreneurial Development. Subsequently, we explained the study’s nature and objectives to the voluntary participants, who provided written informed consent before participating.

(2) A lecture was held using the prepared PowerPoint slides, with all participants attending the lecture session the same time. The lecture lasted approximately 2 h, during which while the slides were presented, the selected song, “Our Father” by Don Moen was played in the background simultaneously in a structured manner. This song was chosen for its slow and soothing nature, with the expectation that it would minimally distract participants (Hallam et al., 2002; Fassbender et al., 2008). The song was played to provide a consistent auditory backdrop continuously throughout all slides of the PowerPoint presentation using external speakers connected to a laptop in a quiet, controlled lecture hall environment with consistent lighting and minimum background noise. Participants were instructed to focus on the content of the slides while the music played in the background. As the song contains lyrical religious content, we believed it might introduce both emotional engagement and semantic interference which would potentially influence attention, comprehension, and memory processes compared to instrumental or non-lyrical music. No interaction with the music was required.

(3) The interview was conducted within 24 h after participants has finished the lectures. This interval was chosen to balance the need for participants to reflect on their experience while minimizing recall bias. However, it is acknowledged that longer intervals could introduce inaccuracies in participants’ recollections of their experiences, as noted in qualitative research standards (Kallio et al., 2016; Lim, 2024; Pannucci and Wilkins, 2010). The interviews took place in a quiet and private setting for about 30–45 min for each participant as recommended by Gill et al. (2008) to ensure that participants felt comfortable and could provide in-depth responses. The interviews were conducted individually, with each participant being asked to share their experiences. The interviews were recorded onto Infinix Smart 8 X6525 smartphone and stored on the first authors laptop for subsequent retrieval and analysis.

### Data analysis

The recorded interviews were carefully transcribed to ensure every word was accurately captured. After transcribing, the data were analyzed using inductive thematic analysis, a method that is commonly used in qualitative research in the absence of theoretical framework (Braun and Clarke, 2020). This approach was chosen because it allows for a flexible and detailed investigation of patterns in the data. The analysis process started with reading the transcripts multiple times to become familiar with the content and understand the overall context (Braun and Clarke, 2020). The first step was to create initial codes by examining the transcripts line-by-line and identifying key ideas or concepts (Braun and Clarke, 2022). These codes were then grouped into related categories, helping to identify broader themes across the data. Next, the researcher reviewed and refined the themes to make sure they accurately reflected the data and were distinct from each other (Nowell et al., 2017). The researcher went back to the data repeatedly to ensure the themes were well-supported by participants’ responses and that they captured the most important points. Finally, the main themes were presented along with direct quotes from the participants to show how the themes were connected to their experiences (Braun et al., 2022). To keep the participants’ identities anonymous, each quote was given a reference number, ensuring their privacy was maintained. This approach helped to make sure the analysis was ethically clear, thorough, and closely tied to what the participants shared.

## Results

After conducting interviews with 23 students, it was found that BM has effects that can be related around three key themes that emerged during the thematic analysis (Table 1). According to Table 1, BM has effects on staying engaged, concentrating, and retention in learning. These themes were used in the discussion to better understand the different ways students experienced the effects of BM during their lectures. Below the table is a more detailed look at each theme, supported by direct quotes from the participants.

**TABLE 1:** Themes, codes, and significant participant statements (verbatim quotes).

Theme	Codes	Significant participant statements (verbatim quotes)
Effects of BM on engagement	Increased engagement Pleasant atmosphere	GS2: “… the slow music goes well with the lesson; it helps me pay more attention to the slides.”
	Improved focus	GS10: “When the music is soft, it makes the class feel nice and helps me think better…….”
	Alertness during lecture	GS5: “The music…… keeps me awake so I don’t think outside class.”
Effects of BM on concentration	Music enhances concentration Music creates calm environment	GS8: “Low music really helps me focus. It makes the lecture hall feel calm and helps me concentrate.”
	Preference for quietness	GS12: “……. I would rather listen to no music. It is easier for me to focus on the lesson when it is quiet.”
	Music as distraction	GS15: “The music was nice, but sometimes I listened more to the music than the lecture.”
Impact of BM on learning retention	Music enhances memory	GS14: “I love it when there is music. It helps me remember things faster. Something about the song makes the lesson stick in my head.”
	Music helps understanding	GS9: “Music makes me remember the lesson better. It feels like the lesson stays in my mind for longer.”
	Music as interference	GS20: “I did not learn much. I just listened to the music and did not realize the class was over.”
	Reduced learning due to distraction	GS3: “I focused more on the music than on what was being taught, so I didn’t learn anything.”

Source: field interview (2024).

### BM and engagement

One of the most common themes was that many students felt that BM helped them stay engaged during the presentation. They reported that the music created a pleasant atmosphere that kept them interested and focused on the presentation. For example, GS2 explained, “……. *the slow music goes well with the lesson; it helps me pay more attention to the slides*.” This suggests that the calm BM helped students focus more on the content of the slides. Similarly, GS10 noted, “*When the music is soft, it makes the class feel nice and helps me think better*…….” The music was seen as a way to make the lecture environment more comfortable and encourage students to think clearly. GS5 also shared, “*The music…… keeps me awake so I don’t think outside class*,” which indicates that the music helped to keep him alert and present during the lecture. These responses show that for many students, BM contributed to them staying engaged and made them more attentive to the material being taught through the PowerPoint slide presentation.

### BM and concentration

On the other hand, the effect of BM on concentration varied among the students. While some found it stimulating their focusing, others felt that it was a distraction. GS8 said, “*Low music really helps me focus. It makes the lecture hall feel calm and helps me concentrate*.” This suggests that for some, music helped create a relaxed atmosphere that allowed them to focus better on the lesson. However, GS12 expressed a different viewpoint: “……. *I would rather listen to no music. It is easier for me to focus on the lesson when it is quiet*.” This shows that some students prefer a quiet lecture presentation environment, feeling that music interferes with their ability to concentrate on the content of slides. GS15 also shared, “*The music was nice, but sometimes I listened more to the music than the lecture*,” indicating that, for some, the music could take attention away from the presentation. These mixed views highlight the fact that while BM may help some students concentrate, others may find it distracting.

### BM and learning retention

The last theme that emerged from the interviews was how BM affected students’ learning and retention or understanding of information. Some students felt that listening to the music improved their ability to understand and remember the lecture concepts, while others disagreed. For instance, GS14 said, “*I love it when there is music. It helps me remember things faster. Something about the song makes the lesson stick in my head.*” This participant seemed to feel that the music helped them retain what was taught in the lecture, with the rhythm of the music possibly aiding memory recall. Similarly, GS9 stated, “*Music makes me remember the lesson better. It feels like the lesson stays in my mind for longer*.” However, not all students had the same experience, some views diverge. GS20 shared, “*I did not learn much. I just listened to the music and did not realize the class was over*.” This participant felt that the music distracted him so much that he lost track of the lecture presentation, which could hinder learning. GS3 also said, “*I focused more on the music than on what was being taught, so I didn’t learn anything*.” These differing perspectives reveal that while some students experience that BM supports learning and memory, others feel that it can be an interference, leading them to become distracted and disengaged from core points presented in the slides.

## Discussion

This study explores the impact of BM on learning during PowerPoint lecture presentations among students in Ghanaian university using a descriptive phenomenological qualitative design. The findings of this study suggest that students experience BM as engaging, but with diverging experiences on the effect on concentration and learning. These findings align with some existing literature while also presenting unique perspectives that diverge from the norm, particularly regarding the nuanced effects of BM on learning and cognition. These results can best be interpreted through the lens of Mayer’s Cognitive Theory of Multimedia Learning (CTML) (Mayer, 2005, 2011, 2024) and de la Mora Velasco (2019) framework, which is an adaptation of Hallam (2012) model of BM in learning contexts.

According to Mayer (2005) in the perspective of the CTML, learning efficiency in multimedia settings depends on how information is processed across the dual channels of visual and auditory input, each of which has limited cognitive capacity. When extraneous stimuli compete for attention, cognitive overload occurs, reducing learning efficiency (Mayer, 2005). The present findings align closely with this principle where most of the participants reported that calm, low-tempo BM helped them sustain focus and improved the atmosphere of the presentation. This indicated that the music functioned as a facilitative affective cue that optimized cognitive load (e.g., Owens and Sweller, 2008). For example, participants who described the music as “pleasant” or “helping them pay more attention” likely experienced reduced extraneous load and enhanced engagement. Conversely, participants who admitted to being distracted or “listening more to the music than focusing on the slides” show how BM can increase cognitive load where it diverts cognitive resources from essential processing. Thus, this duality of the nature of the effects of BM observed here in this study empirically supports the point of Mayer that the design and context of multimedia elements for instruction determine whether they enhance or obstruct learning (Mayer, 2024).

Apart from the cognitive theory of multimedia learning, findings of this study also resonate with de la Mora Velasco’s (2019) adaptation of Hallam’s (2012) framework. According to this adapted framework, the effects of BM on learning can be mediated by personal, contextual, and task-related factors. The diversity of the experiences of the participants in this study as indicated in the interview responses follows this trend. Participants who preferred calm vocal music appeared to benefit more while those whose preferences were not met appeared distracted. This showed the role of personal factors such as musical preference and familiarity in learning under music condition (de la Mora Velasco et al., 2023). Similarly, contextual factors which include the volume and tempo of the BM shaped how students perceived the learning environment, with low volume and softer music described as creating a “calm and comfortable” atmosphere than high volume. Finally, task-related factors played roles in how the outcomes varied among the participants in this study. It can be explained that some participants found the BM to be supportive, that is it provided background stimulator that kept them from getting bored, while others believed that when they needed to think deeply, understand concepts, or connect ideas, the music actually got in the way. This affirms the finding of de la Mora Velasco et al. (2023) that the effects of BM in learning settings cannot be generalized entirely but should be explained with respect to individual learners and the specifics of the instructional task.

Moreover, these findings extend beyond existing theories to the findings of empirical studies. The positive effects of BM on engagement, especially in creating a lively lecture atmosphere for slide presentations, are consistent with previous studies (e.g., Kiss and Linnell, 2021; de la Mora Velasco and Hirumi, 2020) who observed that BM enhances focus or concentration. For example, according to Kiss and Linnell (2021) BM enhances concentration by providing a consistent auditory backdrop, potentially aiding focus during cognitive tasks. Many participants in this study expressed that BM helped them feel more stimulated and connected to the presentation material, which supports research suggesting that music can create an engaging learning environment that encourages active focus (e.g., Griffin et al., 2009). However, this finding contradicts that of Cheah et al. (2022). This research indicates that BM can be a distraction, particularly in tasks requiring complex cognitive processing or intense attention to detail.

This study also highlights the variability in student experiences, as some participants felt that the music distracted them. This reflects the complexity discussed by for example, Echaide et al. (2019), where the effectiveness of BM depends on individual differences. It is clear that the effects of BM may not follow normally as the one-size-fits-all. Hence, lecturers should be mindful if they intend to augment their PowerPoint presentations with BM; they should also consider students individual and personality differences when selecting music for lectures presentations.

In terms of concentration, while many students found that the music been calming, soft was helpful in maintaining focus, others expressed concerns about distraction, which echoes findings by Thompson et al. (2011). This divergence in experience suggests that student concentration is influenced by personality, which should be taken into account when deciding whether to incorporate music into lectures or not. Future research could explore how different types of music affect concentration and learning in different types of personalities, as well as how these effects differ based on individual student preferences and learning environment.

The effects of BM on learning outcomes showed that some students felt that music helped them with memory retention and understanding, supporting findings from Guo et al. (2021). This suggests that music can be an effective tool for cognitive enhancement when appropriately used. However, not all participants shared this view, with some feeling that the music distracted them from the content. This disparity points to the need for further research to quantify the actual impact of BM on learning in a similar setting. Specifically, studies should consider different genres or tempos of music and explore how they may influence retention, comprehension, and overall academic performance during PowerPoint presentations. Additionally, it would be valuable to examine how contextual factors such as the type of content being taught or the student’s previous exposure to music in educational settings may impact the effectiveness of BM.

### Limitations and suggestions for future research

One limitation of this study is the reliance on self-reported data that could introduce bias, as students may perceive their learning experiences differently from how they are actually affected by the background music. Future research could address these limitations by employing a combination of self-report and observational data. Also, future studies might use different qualitative designs such as narrative phenomenology or adopt mixed methods designs for the sake of getting triangulated insights that go beyond those provided by the present study. Furthermore, longitudinal studies could help determine the long-term effects of BM on student learning outcomes in PowerPoint presentations. The contrasting views as found in this study highlight the need for further research to better understand how different types of students respond to background music in learning environments. It may be that individual preferences, personality traits, or learning styles influence whether BM enhances or disrupts learning. Therefore, it is important to explore how factors such as music type, volume, and timing may affect students’ learning experiences in lecture slide presentations.

## Conclusion

This study explored students’ experiences of BM effects in PowerPoint presentations in a lecture setting. The findings suggest that BM, particularly calming and soft music, can enhance student engagement by creating a more dynamic and stimulating learning environment. However, the effect of BM on concentration varied, with some students reporting that it improved their focus, while others found it distracting. These mixed views highlight the importance of considering individual preferences when integrating music into lectures. Additionally, while many students believed that background music helped with retention and understanding, there were concerns about its potential to disrupt learning. In a nut shell, this study emphasizes the need for lecturers to be mindful of the diverse needs of their students when using BM and suggests that a tailored approach may be most effective in enhancing learning experiences. Also, this study concludes that, lecturers should remain aware that BM is just one tool among many, and its effectiveness will depend on various factors such as the type of lecture presentation material and the learning environment.

## Data Availability

The raw data supporting the conclusions of this article will be made available by the author, without undue reservation.

## Footnotes

1
https://www.youtube.com/watch?v=ajMqhFz3FIk
